# Utilizing Zebrafish Visual Behaviors in Drug Screening for Retinal Degeneration

**DOI:** 10.3390/ijms18061185

**Published:** 2017-06-02

**Authors:** Logan Ganzen, Prahatha Venkatraman, Chi Pui Pang, Yuk Fai Leung, Mingzhi Zhang

**Affiliations:** 1 Department of Biological Sciences, Purdue University, 915 W. State Street, West Lafayette, IN 47907, USA; lganzen@purdue.edu (L.G.); pvenkatr@purdue.edu (P.V.); 2 Department of Ophthalmology and Visual Sciences, Chinese University of Hong Kong, Hong Kong, China; cppang@cuhk.edu.hk; 3 Department of Biochemistry and Molecular Biology, Indiana University School of Medicine Lafayette, 625 Harrison Street, West Lafayette, IN 47907, USA; 4 Purdue Institute for Integrative neuroscience, 610 Purdue Mall, Purdue University, West Lafayette, IN 47907, USA; 5 Purdue Institute for Drug Discovery, 610 Purdue Mall, Purdue University, West Lafayette, IN 47907, USA; 6 Joint Shantou International Eye Center, Shantou University & the Chinese University of Hong Kong, Shantou 515041, China

**Keywords:** zebrafish, visual behaviors, visual motor response, drug screening, retinal degeneration, retinitis pigmentosa, night blindness

## Abstract

Zebrafish are a popular vertebrate model in drug discovery. They produce a large number of small and rapidly-developing embryos. These embryos display rich visual-behaviors that can be used to screen drugs for treating retinal degeneration (RD). RD comprises blinding diseases such as Retinitis Pigmentosa, which affects 1 in 4000 people. This disease has no definitive cure, emphasizing an urgency to identify new drugs. In this review, we will discuss advantages, challenges, and research developments in using zebrafish behaviors to screen drugs in vivo. We will specifically discuss a visual-motor response that can potentially expedite discovery of new RD drugs.

## 1. Retinal Degeneration Overview

Retinal degeneration (RD) is a leading cause for blindness in humans [1,2]. It encompasses several inherited retinal dystrophies, such as retinitis pigmentosa (RP) and Leber congenital amaurosis (LCA), age-related macular degeneration (AMD), and syndromes that affect eyes. RD can be broadly classified into three types: (i) diseases primarily affecting rods; (ii) diseases primarily affecting cones; and (iii) diseases where both photoreceptors (PRs) are affected (for a comprehensive review, (see [2]). The severity of these diseases varies. Some of them are stationary, while others are progressive and present with symptoms at adulthood. Regardless of the etiology and pathogenesis, when the light-sensitive PRs die, they cannot properly transmit visual signals. Consequently, the patients lose some of their vision, or even become blind.

To illustrate the complexity of RD, here we will consider two subtypes: RP and LCA. RP is a group of genetically heterogeneous diseases affecting 1 in 4000 people, in both the US and the world [3]. It has more than 45 causal mutations and different modes of inheritance. Most RP cases are autosomal-recessive (50–60%), while the others are autosomal-dominant (30–40%) and X-linked (5–15%) [1]. These patients experienced reduced vision in dim light or night blindness due to rod degeneration. These dying rods progressively affect the cones in the macula, ultimately leading to blindness [1,4]. LCA has a birth prevalence of two to three cases in 100,000 and is attributed to more than 5% of all retinal dystrophies [1,5,6]. It has more than 14 associated disease-causing genes. Some of them are expressed in the PRs, whereas some others are expressed in the retinal pigment epithelium (RPE) that lays below the retina. The mode of inheritance is mostly autosomal recessive, but autosomal dominant inheritance is also possible. The LCA patients suffer from severe visual impairment or blindness, and photophobia [6]. These disease examples highlight the severity and complexity of manifestation of RD, which is often caused by genetic heterogeneity.

Many RD mutations affect proteins in the phototransduction pathway, or the well-being of the retinal cells. In the former situation, the mutations lead to either absence or failure to form correct proteins, and disrupt the phototransduction pathway. This severely affects PR physiology, leads to their degeneration, and eventually causes blindness in patients. For example, mutations in *RHO* (rhodopsin) and *PDE6B* (rod-specific phosphodiesterase subunit) are linked to RP [7,8,9]. The patients experience tunnel vision, constricted visual fields, and progressive decline in vision. Similarly, mutations in *GNAT1/GNAT2* (rod and cone transducin subunits) are found in patients with severe night blindness and achromatopsia [2,9]. Mutations in *PDE6C* (a cone-specific phosphodiesterase subunit) are found in patients with cone dystrophy or achromatopsia. They experience progressive decline in visual acuity and eventually lose color vision [10,11,12,13]. In some other situations, RD mutations affect the well-being of retina. As discussed above, LCA is a congenital condition that can affect genes expressed in either PR or RPE. The condition is commonly associated with *RPE65* and *LRAT* that are specifically expressed in the RPE. These RPE-expressed genes play a critical role in isomerizing the all-*trans* retinol to 11-*cis* retinal and regenerating the visual pigment. Their mutations compromise the physiology of PRs and ultimately lead to their degeneration. These examples indicate how multiple mutations in different genes can affect PR physiology in different ways. It is therefore not surprising that RD’s severity and progression vary in different cases—some forms are early onset, whilst some others are late onset. This genetic heterogeneity not only emphasizes the importance of understanding etiology and pathogenesis of RD, but also the challenges of finding specific treatments for different conditions.

## 2. Current and Experimental RD Treatments

There are few treatment options for most RD patients. In patients suffering from wet or exudative AMD, their retinal blood vessels leak and overgrow. The overgrown vessels detach retina and result in visual impairment. These patients are currently treated by anti-angiogenic drugs (e.g., anti-VEGF, a growth factor that promotes vessel growth), and surgery, like photodynamic therapy and photocoagulation, to seal the leaky blood vessel [14]. For many congenital RD patients, there is no treatment. To find new therapies, researchers are working on several fronts, including gene therapy [15,16,17], stem-cell therapy [18,19,20], retinal transplantation [21], optogenetics (a way to confer light sensitivity to other retinal cells through expression of light-sensitive proteins called channelrhodopsin) [22,23], prosthetics [24,25], and new drug discovery. In gene therapy, a functional copy of the gene is introduced to the target cell type in the disorders with loss-of-function mutations. Alternatively, the mutated gene in autosomal dominant disorders are suppressed or replaced. In some RP mice models, PRs might die of oxidative stress. This oxidative damage could be reduced by introducing genes encoding super-oxide dismutase and glutathione peroxidase. This approach extended cone survival in these RP models [26,27]. With stem-cell therapy, stem cells or induced pluripotent stem cells [28,29] are used to derive replacement cells for tissues damaged by RD, including PRs and their precursors, and RPE [30,31,32,33]. In retinal transplantation, healthy neurons are transplanted into diseased retinas. For example, PR sheets harvested from human cadavers were transplanted to late-stage RP patients [21]. Optogenetics re-introduces light sensation to the residual retina by expressing different types of opsins (rhodopsin, melanopsin or channelrhodopsins) [23,34,35,36]. For patients with advanced RP, prosthetics may be a viable option, akin to the hearing aid for those with hearing impairment. The Argus II retinal prosthesis system is the only FDA approved prosthetic system available [24,25]. In a 3-year follow up study, it improved visual acuity and visual function in 30 patients, without affecting their quality of life [25]. Despite these encouraging results, the cost to implant an Argus II is about USD $14,988 per eye. Its affordability is debatable, even though it may be cost effective when compared to long-term usual care [37]. For drug discovery, many studies have evaluated the effects of vitamin A supplementation [38,39] and docosahexaenoic acid (DHA) [40,41], with conflicting observations. Others have studied growth factor supplementation, treatment with anti-oxidant compounds [27,42,43], and naturally-derived compounds with therapeutic potential. For example, when two RP mouse models were treated with anti-oxidants, they experienced a reduction in the death rate of cones [42,43]. Even though these treatment options may be promising, they are largely experimental. We are in need of therapeutic interventions which are not only potent, but also cost-effective. This need can be met by screening drugs in animal models that can be efficiently evaluated. Several animal models are used in vision research, ranging from invertebrate fruit flies, to vertebrate animals, such as zebrafish and rodents. Each of these models has its own strengths and weaknesses, and is suitable for addressing different research questions [44]. Among these models, zebrafish are particularly suitable for screening eye drugs, as their visual system is very comparable to that in humans.

## 3. Zebrafish as a Model System for the Eye

The zebrafish (*Danio rerio*) has become an extremely popular and useful animal model in modern research. Compared to other animal models, zebrafish offer numerous advantages, including high fecundity, amenability to genetic manipulation, ease of handling and maintenance, low costs, relatively transparent embryos, and similarity to mammals [45]. In stark contrast to mice, a single breeding pair of adult zebrafish can produce 100 to 200 embryos on a weekly basis. The ample supply of embryos facilitates high-throughput in vivo experiments, and large-scale screens that would otherwise be inefficient to perform.

Zebrafish eyes have important similarities, and some differences, to the human eyes. For example, the zebrafish have predominantly cone-mediated vision [46] like humans. Humans possess three types of PRs for color vision: red, green, and blue cones; the zebrafish contain the same PR types, and also an ultraviolet cone [47]. Additionally, red and green cones in zebrafish are double cones, rather than individual cones. Zebrafish also possess rods. Thus, they perceive light through similar cellular mechanisms as in humans. The retinas of zebrafish and humans also share the same layout, with three cellular layers: the outer nuclear layer (ONL), the inner nuclear layer (INL), and the ganglion cell layer (GCL). They also have two synaptic layers: the inner and outer plexiform layers (IPL and OPL). Zebrafish and humans also both contain RPE. However, the zebrafish retina differs from the human retina in some important aspects. For one, zebrafish eyes are positioned more laterally, while human eyes are located frontally on the face. Zebrafish also lack a macula and fovea. Instead, their photoreceptors are organized in a mosaic pattern throughout the retina [48,49,50]. In contrast, humans have a macula in which cone density is highest. The density dramatically decreases towards the periphery, where rod density is highest [51,52]. These features of zebrafish retina uniquely positions zebrafish as a suitable model for vision research. Other popular models include rodent models, such as the mouse and rat. Rodents have evolved to live a primarily nocturnal lifestyle, and hence utilize rods for their vision [53]. They are also dichromatic and only possess short and medium wavelength cone PRs. Their vision therefore is very different from the trichromatic humans who follow a diurnal lifestyle. Rodents also lack calycal processes in photoreceptors which are found in human and zebrafish photoreceptors. Even though these rodent models have revealed critical insights into visual sciences, they may not always be the ideal system for research on human vision. In contrast, the tetrachromatic and diurnal zebrafish more closely match the vision and evolved lifestyle of the humans. Thus, they may be more suitable for modeling human retinal diseases.

The zebrafish have been used to model RD [54,55,56,57,58]. As discussed before, this heterogeneous disease is caused by mutations in various genes. For example, RP is often caused by mutations in *RHO* [1], which encodes the photopigment in rod PRs for initiating phototransduction. One clinically-relevant zebrafish RP line expresses a human *RHO* transgene with a truncation mutation Q344X [59]. This Q344X mutation was originally identified in patients who suffered from autosomal dominant form of RP. Another prominent model of RD is the *pde6c* zebrafish mutant [60,61]. It carries a splice-site mutation in *pde6c*, a subunit of cone-specific phosphodiesterase, which results in a truncated protein. The mutation leading to cone degeneration in a mechanism seems to be analogous to the *Pde6b* mutation in the *rd1* mouse [60,62]. Thus, zebrafish can be used to model clinically-relevant RD. These models can also be used to identify new drugs, using approaches that allow for large-scale discovery.

## 4. Zebrafish for Large-Scale In Vivo Drug Screening

Zebrafish have been used in a number of seminal studies to screen for compounds with therapeutic effects [63,64,65,66]. For example, Rihel et al. utilized the wake/rest behavior of larval zebrafish to screen over 5600 compounds in search for molecules that altered the behavior [67]. Recently, several studies have utilized zebrafish as an in vivo model to discover potential new drugs (Table 1). This indicates a shift in interest towards in vivo drug discovery. As an example, Kokel et al. used the photomotor response and the touch response of zebrafish embryos to screen over 14,000 chemicals to identify neuroactive compounds [68]. These pioneering studies show that zebrafish behaviors can be utilized as phenotypes for screening neuroactive compounds, and can potentially be used for screening drugs for RD. However, it should be noted that zebrafish larvae are still undergoing development during the drug screening process. Thus, it may not be feasible to study some drug or disease mechanisms with zebrafish larvae.

To date, all drug-screening methods have yet to produce any viable treatments for RD patients. Phenotype-based screening using larval zebrafish behavior may provide the method needed to discover new drug leads. Behaviors are the processed outputs of a perceived stimulus. In the case of vision, zebrafish exhibit a number of visually-mediated behaviors upon different light stimuli [79,80,81,82,83,84,85]. When these fish carry mutations that affect the visual system, their visual behavior may be altered or ablated. This altered behavior provides the necessary phenotype to design screens that find compounds to ameliorate the abnormal behavior [86]. Leading compounds identified through this process may have a higher likelihood of efficacy, because their therapeutic effects are seen functionally on the systems level, and not only at the cellular or molecular level (for a comprehensive review of zebrafish behaviors and visual behaviors, see [86,87,88]).

## 5. Zebrafish Visual Behaviors

### 5.1. Optokinetic Response (OKR) and the Optomotor Response (OMR)

The optokinetic response (OKR) is one of the visual behaviors that can be observed in zebrafish larvae as early as 3 days post fertilization (dpf) and robustly starting at 5 dpf [84,89,90,91]. In this assay, the larvae are immobilized in a viscous solution, such as methylcellulose, and then presented with a visual stimulus. The stimulus is usually a set of black and white stripes rotating clockwise or counterclockwise around the fish. This rotating grating elicits a stereotypical behavior in normal larvae, consisting of eye movement along the direction of rotation of stripes and saccades. If the larvae have visual impairments, they may display abnormal OKR. Therefore, OKR can be a good tool for evaluating visual function.

The optomotor response (OMR) is another visual behavior that zebrafish exhibit beginning between 6 and 7dpf [88,92]. A broad moving stimulus is presented below the larvae, which induces a swimming behavior in the direction of the stimulus. This can be achieved by placing a monitor below a tank of larvae to present the stimulus, and swimming behavior can be captured with a camera, and quantified. The OMR behavior is a result of the larvae attempting to counter water currents and remain in place. As this behavior is visually modulated, the OMR can be used to generally assess the vision of zebrafish larvae. Larvae that do not have functional photoreceptors or retinal ganglion cells do not exhibit an OMR [81].

The OKR and OMR have long been used in forward-genetic screens to identify visual mutants [81,84,93]. In such screens, parental fish are subjected to chemical mutagenesis which would create random mutations in the genome in the gametes. If the mutation hits a critical gene for vision, the progeny of these mutagenized adults may display abnormal OKR and OMR. By systematically analyzing the OKR of many progenies, many visual mutants have been identified. For example, Brockerhoff et al. used OKR to evaluate 266 mutagenized genomes and isolated 18 OKR mutants [84]. Neuhauss et al. used the OKR and the OMR assays to assess visual defects in 450 previously created mutants [81]. They were able to identify and characterize visual defects in 13 mutants. The visual mutants identified from these studies revealed new insights into cellular and molecular defects that lead to different types of visual problems. For example, the *no optokinetic response a* (*noa*) mutant lacked OKR, and displayed an abnormal electroretinogram (ERG) in which b-wave had a delayed onset and a smaller amplitude, whereas a-wave was fairly normal [84]. The mutation was subsequently mapped to *dihydrolipoamide S-acetyltransferase* (*dlat*), a subunit of pyruvate dehydrogenase (Pdh) [94]. The Pdh deficiency likely affected neurons including PRs, that demand high energy, and resulted in the OKR defect of *noa* mutants. In recent years, the OKR has also been used in reverse-genetic studies that revealed visual functions of genes that were mutated in patients [95]. These examples illustrate the power of using OKR and OMR to isolate visual mutants and dissect the genetic-basis of the defects.

More recently, OKR has also been used to evaluate the efficacy and toxicity of drugs. For example, it was used to test the effects of two anxiolytic drugs—lorazepam and diazepam [96]. These two benzodiazepines caused zebrafish larvae to spend more time in the dark during light/dark presentation, compared with the vehicle-treated controls. This aversion behavior was not likely caused by drug-induced defects on the visual system, as the compounds did not affect OKR. Even though this study did not focus on RD, it demonstrates the power of OKR in toxicology research. The OKR has also been used to evaluate oculotoxicity in a seminal study, in which WT larvae were treated with six known oculotoxic drugs, and the resulting effects were assayed by two visual behaviors: OKR and the VMR (visual motor response, see next section) [97]. Both assays showed that the tested compounds interfered with OKR and VMR—hence, these compounds were indeed oculotoxic. These behavioral assays can therefore be used to detect oculotoxic drugs. In addition to the OKR, the OMR has also been used to been used to evaluate the oculotoxicity of drugs. Richards et al. studied the effects of 27 compounds on OMR utilizing zebrafish larvae [98]. The study was able to utilize the OMR to correctly identify oculotoxic compounds 70% of the time. These studies show that the OKR and the OMR can be used to evaluate the drug effects on vision.

Even though OKR has been used in toxicology and pharmacology studies, its slower throughput may hamper its utility in high-throughput drug screening. Only ten or fewer larvae can be reliably tracked at the same time using automated-tracking system, because they may move in the immobilizing solution. Realigning these larvae can become tedious and laborious, and create a bottleneck in drug screening. Instead, the OKR should be used for the high-content analysis of identified drug candidates. The assay can analyze many parameters of a sample, such as rotation velocity and spatial frequency of the grating, and color of and contrast between the stripes in the grating [99]. Measuring these fine parameters can reveal how a candidate drug may have improved visual function. Therefore, the OKR may be used as a secondary assay to characterize identified drugs, but not the primary assay for high-throughput drug screening. To this end, we need an assay that can be done high-throughput.

### 5.2. Visual Motor Response (VMR)

**Figure 1:**
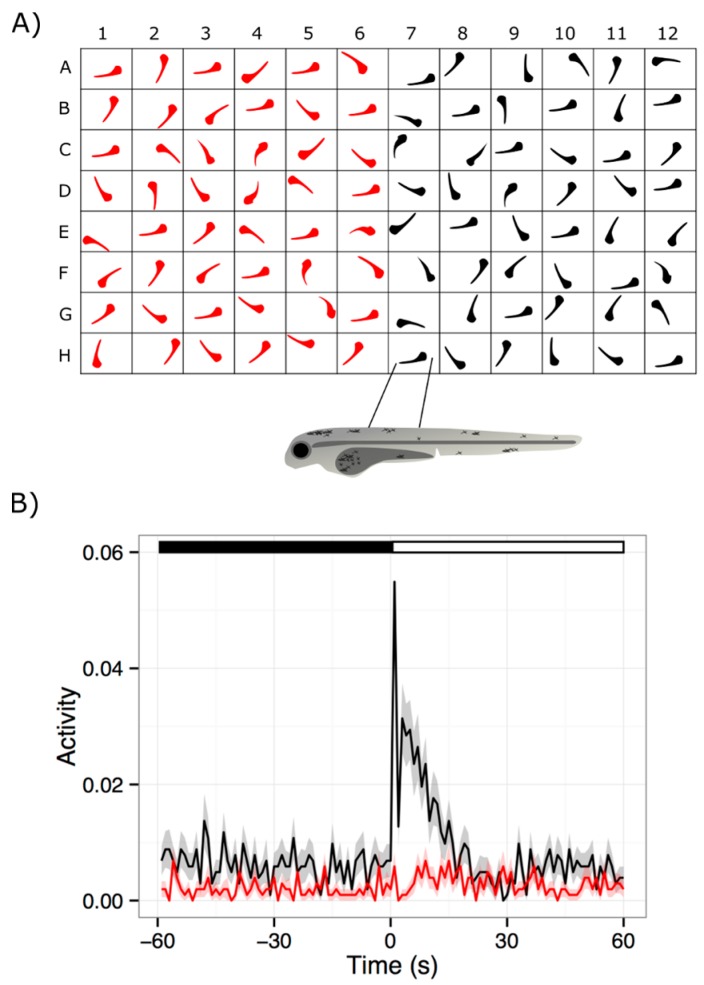
A typical visual motor response (VMR) experiment. Sufficient zebrafish embryos for an experiment are collected by breeding adult fish. The embryos can be maintained in petri dishes with media, as they develop until they are needed for a VMR assay. (**A**) At the appropriate stage, embryos can be placed into 96-well plate format to facilitate throughput, storage and, data collection during a VMR assay. It should be noted that there are multiple arrangements possible for placing zebrafish larvae in a 96-well plate, such as row-wise, column-wise, or checkerboard patterns. Larvae in the 96-well plate arrangement can then be placed in a light-proof recording chamber and exposed to light onset or light offset stimulus. The locomotor output of the larvae is recorded and processed. Recorded data can be visualized through programs such as R 3.4.0 [123] (**B**) This graph illustrates the VMR of a group of 7-dpf wild-type larvae (black trace) responding to light onset stimulus [106]. Their response is compared to the VMR from a group of visually-impaired *pde6c* mutant larvae (red trace). Healthy larvae exhibit a strong startle response to the light onset, while the visually-impaired larvae do not. This lack of response by retinal degeneration (RD) zebrafish models forms the basis for drug screens.

The visual motor response (VMR) is a startle response mediated by vision, and initiated by a drastic change in illumination [94,95]. It can be detected in larvae as early as 3 dpf, and becomes robust by 5 dpf [80,100,101]. In a typical VMR assay, larvae are individually placed in a multi-well plate, which is then placed inside a lightproof machine to isolate from external disturbances and environmental light. These larvae are stimulated with controlled white light, and then their resultant movements are simultaneously visualized by infrared (IR) illumination and recorded with infrared camera in the machine. The basic workflow of this process is outlined in Figure 1. Similar VMR systems are currently offered by Viewpoint LifeSciences [102] and Noldus [103], or are homemade [104,105]. Regardless of the system, the collected VMR generally has two separate parts—a response to drastic light onset (Light-on VMR), and a response to drastic light offset (Light-off VMR) [80,101,106]. The Light-on VMR of WT larvae consists of a drastic and sharp increase in locomotor activity at light onset, followed by a rapid decrease in activity to the baseline level after approximately 30 s. Similarly, the Light-off VMR of WT larvae consists of a drastic increase in locomotor activity at light offset. However, unlike Light-on VMR, the Light-off VMR does not immediately return to the baseline level in 30 s, but rather sustains at an intermediate level. After 30 min, this activity gradually returns to the baseline level. The standard assay may have several alternating light and dark phases, so that the larvae will be subjected to multiple trials of light onset and offset. This standard assay is usually performed with 96-well plates, but other formats have also been used [104,107,108]. Since the well dimensions are known to affect the locomotor behavior [107], it is advisable to use one type of plate for a project. Here, we will restrict our discussion on the information we learned from larval activities collected from 96-well plates.

The larval activity in the VMR is generally summarized in larval movement per unit time [67,79,80,97,100,101,106,109] and displacement [110,111,112,113,114,115,116,117]. In the former method, each larva is registered in each frame of the video as pixels. These registered pixels are compared with those in the next frame. If a larva moves, the corresponding registered pixels will move too. Larval movement can therefore be detected by counting the number of registered pixels that change beyond a pre-defined threshold in successive frames. This frame-by-frame movement can be averaged over a specific timeframe, which allows for larval movement per unit time. By selecting appropriate thresholds, larval activity can be further categorized into different levels, for example: large, medium, and small/no movement [67,97,100,109]. This categorization may help reveal different types of locomotor outputs in response to different influences, including drugs and genetic variations. For example, our lab found that using small movements of larvae in machine learning enhanced the accuracy in classifying different WT strains [109]. This observation suggests that the difference in small movements between WT strains was originated from their genetic variations. On the other hand, the displacement method compares the registered pixels between successive frames in a slightly different way. It measures the actual distance between the centroids of the registered pixels between successive frames. This approach allows for the calculation of velocity and reveals different aspect of larval locomotion. These functionalities are readily available in the software from the commercial systems; similar functions are often available in homemade software for tracking zebrafish locomotion [104,118,119,120,121,122]. Regardless of the summarization approach, the VMR activity is often presented as the average of multiple larvae of the same type, or under the same treatment. Since this activity reflects the neural output of the larvae upon light stimulation, the VMR assay can potentially be used to evaluate drug effects on the visual system.

The VMR is conducive to high-throughput screening of drugs for RD because: (i) it can analyze 96 larvae and several drugs at once in one plate; (ii) it is simple to set up and assay, and is less laborious compared to other visual analyses; (iii) it tests in vivo effects of drug treatments; and (vi) it allows for simple drug administration, by mixing the drug with fish water. These advantages make it possible to firstly screen and identify drugs that may enhance light sensation, and then to study the mechanism of positive drugs. For example, we recently used the VMR to identify a compound that may treat RD [106]. We found that a RD mutant *pde6c* displayed significantly reduced VMR compared with the WT, and that the reduced VMR was ameliorated upon by exposure to schisandrin B, a naturally-derived compound. We then found that this compound reduced the abnormally-large rods, but did not exert any measurable effects on cones. Therefore, the compound might have exerted its effect on VMR through a beneficial effect on the rods. As mentioned in the previous section, the VMR was also used for evaluating oculotoxicity of six known oculotoxic drugs, which is essentially a drug screen [97]. This study concluded that for the visual-toxicity test, the VMR has a sensitivity of 83%, a specificity of 90%, and a positive predictive value of 83%. Together, these initial characterizations strongly indicate that the VMR can facilitate high-throughput drug discovery.

## 6. Current Issues and Developments of VMR for Drug Discovery

Since VMR has shown great promise in drug discovery for RD, we must consider the ongoing developments and its several limitations. In this section, we will consider seven issues related to the neural basis of VMR, assay optimization and development, and data analysis.

### 6.1. Extraocular PRs and Locomotor Response

We defined earlier that VMR is a visual startle that is initiated by a drastic light change. Depending on the assaying parameters, the VMR assay may also detect light sensation by extraocular PRs. This extraocular-PR contribution was deduced by two observations: (1) an eyeless *chokh/rx3* mutant lacks VMR [79]; and (2) eye enucleation abolished VMR [106]. However, these deductions were also based on VMR conducted with the original protocol established by Emran et al., which summarized larval activity in bins of seconds [79,80]. When the *chokh* mutants were subjected to a similar assay, and their activities summarized in bins of minutes, they displayed a delayed locomotor activity during light offset in a scale of minutes [116]. Subsequent analysis reviewed that this delayed locomotion was driven by the deep-brain PRs of the eyeless *chokh/rx3* mutants. Based on these observations, we recommend the following design to maximize detection RD drugs by the VMR assay: (1) use the original VMR protocol [80,106] that summarizes activity in seconds to screen drugs for RD; (2) focus on analyzing the initial seconds of the VMR after light change, up to 20 s; and (3) test any positive candidates against the eyeless *chokh/rx3* mutants, or enucleated larvae, with the original VMR protocol to exclude drug effects originating from extraocular PRs.

### 6.2. Neural Basis of VMR

**Figure 2:**
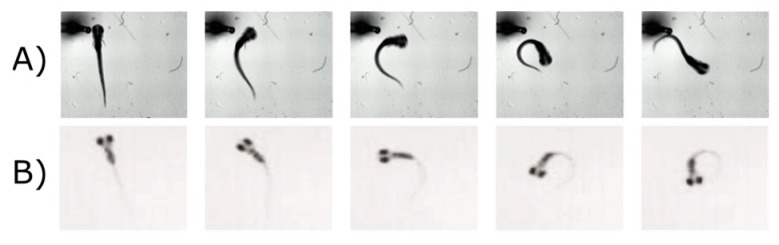
Zebrafish larvae display different startle escape behaviors. (**A**) A larva escapes from a touch-stimulus by exhibiting a C-bend. In this response, larvae curve their bodies in a C-shape and swim quickly away from the location of the stimulus [128]; (**B**) Larvae orient into an O-bend in response to a dark flash. The larva curves its body approximately 180 degrees to swim in the opposite direction [125]. Reproduced with permissions from Burgess et al. and Lorent et al.

The underlying brain circuitry that drives VMR is unclear [87], as also illustrated in the last section. The VMR is a startle response, which is usually activated by a number of hindbrain reticulospinal neurons [124]. The exact neurons driving the VMR are not clear, and we have only recently begun to learn the circuitry that drives locomotor behavior during light offset through two studies [116,125]. When zebrafish larvae were stimulated by dark flash, they twisted their body to form a circular shape that was termed the O-bend (Figure 2B). This O-bend was distinct from a less drastic version of body twist, termed the C-bend (Figure 2A), which was initiated by the Mauthner (M) cells in the hindbrain [126]. When the M-cells were ablated, the larvae could still display an O-bend upon a dark flash [125]. Interestingly, the O-bend was abolished by eye enucleation, suggesting this initial dark response was initiated by retina and was independent of M-cells [116]. Future studies should focus on dissecting the roles of different reticulospinal neurons in different parts of VMR [124]. This can be achieved by systematically ablating reticulospinal neurons, and measuring the VMR in the ablated larvae [125,127].

### 6.3. Rod and Cone Responses in VMR

The VMR does not directly measure pure rod and cone responses at the moment. The basic setup from commercial suppliers uses white LEDs as the light source, and does not necessarily discriminate rod and cone contributions. We and other colleagues have been addressing these issues. In our preliminary studies, we detected VMR from a cone mutant *nof*/*gnat2* that lacked functional cones but possessed functional rods [129], suggesting rods contribute to the VMR [130]. We also introduced neutral-density filters to the light path of the VMR machine, and still detect appreciable VMR from WT larvae at light intensities that drive scotopic vision (unpublished observations). These studies suggest that the VMR assay can detect rod response. To discriminate responses driven by different colour cones, the VMR machine can be modified to use LEDs with wavelengths matching the absorption maxima of respective cones. Such a machine has been built by Noldus and several groups [105,131]. In one of these studies [131], the machine has been used to test VMR driven by different wavelengths, which begin to reveal different cone contributions to VMR

### 6.4. VMR Assay Optimization

The assaying time for VMR can be optimized. In the original protocol established by Emran et al. [80,101], the total run time is approximately 6 h. This protocol involves a dark adaptation for at least three hours, and three trials/technical repeats of light onset and offset, with each light change lasting for 30 min. For larger-scale screening, this long protocol should be streamlined. First, the long dark adaptation may be shortened without affecting VMR initiation. Even though using three technical repeats may confer some analytical advantages during initial screens, they do not replace biological repeats which address biological variations. We also found that the first technical repeat of Light-on VMR was significantly different from the second and third technical repeats, whereas all three technical repeats of Light-off VMR were comparable [101]. Therefore, we may also shorten the VMR assay by using just one trial of light onset and offset, and focus on generating biological repeats when necessary. Together, these modifications may shorten a VMR assay to approximately 1.5 h. Other colleagues have used different time periods in their VMR trials. For example, Mora-Zamorano et al. used a 10-min dark adaptation, followed by two cycles of 10 min light-on and light-off cycles. Their run time was 50 min [132]. Deeti et al. used a variation in which the light was turned on for the first 30 min, followed by four on-off cycles in 20 min intervals [97]. Their assay time was 1 h and 40 min. For screening eye drugs, we recommend the length of each light onset and offset period should be maintained at 30 min. In each light period, the larvae are essentially adapting to light or darkness. Shortening this adaptation may affect the VMR of subsequent light change. For example, zebrafish larvae require 20–30 min light adaptation before they would maximally responsive to dark flash and display the characteristic O-bend [125], as discussed above. In addition, we could not detect a drastic Light-on VMR when the previous dark phase was shorter than 30 min, probably due to the larvae being still quite active in the dark phase until the end of the 30-min period (unpublished observations). When the underlying circuitry for VMR is fully elucidated, we can potentially further optimize the assay design and shorten the assaying time.

### 6.5. VMR of Adult Zebrafish

VMR is currently performed using larvae, and may be more suitable for screening drugs for early-onset RD [133]. In this sense, OKR may be more suitable for screening drugs for late onset retinal diseases, as it can be performed on adult fish. Nonetheless, adult zebrafish also display visual startle, and can be tracked in parallel by advanced computer vision [134]. We therefore believe that the VMR can also be adapted for studying adult-onset RDs, which in turn will expand our capability in finding new drugs for these conditions. However, the throughput of studying VMR in adults is likely lower. Despite lower throughput, this adaptation would be suitable for testing promising candidate compounds identified by other higher throughput screens.

### 6.6. Data Analysis of VMR

VMR data are complex and require new approaches for data analysis. The complexity of VMR data comes from the experimental design, of which there are a few challenges, which we will highlight here. First, in a VMR experiment, multiple larvae are measured repeatedly over a long period of time. These repeated measurements are correlated in time (time-dependent), and cannot be handled by t-test and ANOVA. These tests also compare data at a specific time point. They need to be run multiple times to analyze the multiple time points in the time-series data. This would increase the overall type I error rate, and the probability of rejecting the null hypothesis when it is true (i.e., false positives). This time-dependent issue is often handled by repeated-measures ANOVA, a variant of ANOVA for analyzing repeated measurements. Nonetheless, this test requires data variance satisfies sphericity assumption, which stipulates that the variances of the differences between group combinations are equal. To handle these analytical issues, we recently introduced Hotelling’s *T*-squared test for analyzing locomotor data [101]. This test not only reduces the type I error rate compared to the t-test, but also takes into account the time dependency between repeated measures. It allows for comparing the activity profile of two groups of samples in a specified timeframe, and gives an intuitive p-value for significance inference. Second, summarization of larval activity can take place in short time bins (e.g., seconds), during which many larvae may displaying little or no movement. As a result, the distribution of larval activities would likely deviate from a Gaussian distribution. This deviation creates data imbalance and poses challenges to traditional analyses which rely on the assumption of a Gaussian distribution. To address this issue, we introduced another approach—the logistic generalized linear mixed model (logistic GLMM) [135]. This approach handles binary response variable, and can be used to estimate the probability of the binary response based on multiple predictors. It also assumes that the conditional distribution of the response variable is a Bernoulli distribution rather than a Gaussian distribution. When we applied this approach, we transformed the activity values into binary responses as 0 (no movement) and 1 (otherwise). This transformation made the data less imbalanced. The logistic GLMM also addresses another issue in repeated measurements, in which the larvae from the same location of the 96-well plate tend to correlate over time. This issue is handled in the logistic GLMM by treating group-level terms, such as location, as random effects. These effects can be used to characterize the correlation between observations in the same group. We believe these new analyses complement each other and effectively analyze VMR data. Other colleagues have also visualized VMR data with different approaches, including heatmap [136], mapping the movement back to individual wells of the 96-well plate [104], or creating web-based application to process raw data and perform standard statistical tests [137].

### 6.7. Determining Sample Size for VMR Analysis

An appropriate number of larvae must be chosen in each experimental group for efficient VMR analysis. To this end, we conducted power analysis of the Hotelling’s *T*-squared test, and determined several factors for selection appropriate sample size [101]. In general, a small number of samples are needed for the test to attain statistical significance. This number is a function of the length of time period used in the analysis, effect size, and the number of experimental repeats. In most cases, the number is compatible with the 96-well plate format. For example, only 32 larval samples are needed in each group, if the time period is two seconds, and the effect size equals to 0.8. The actual number can be proportionally reduced with appropriate experimental replications. The best type would be biological repeats; the other type would be technical repeats. As discussed above, the original VMR protocol consists of three technical repeats of light onset and offset. If used cautiously, these technical repeats can reduce the number of larvae needed for each group. However, using data from technical repeats may suffer from pseudoreplication, a scenario when the repeats are not independently measured. This would increase type I error rate and reduce confidence intervals. In addition, we also found that not all technical repeats can be combined because we identified difference between technical repeats in the original VMR protocol [101]. As stated above, the first Light-on response was statistically different from the second and the third; whereas all three Light-off response were comparable. This difference was probably due to the longer 3.5-h dark adaptation prior to the first Light-on response, while all other technical repeats were preceded by the same 30-min light/dark phase. Nonetheless, these observations indicate that the first Light-on response should be analyzed separately, and the other repeats of the same type may be combined. Therefore, we recommend caution when using technical repeats, which are better restricted for a first-pass screen. The positive findings should be validated by biological repeats and follow-up experiments. Future studies should also focus on testing additional approaches to reduce sample number to facilitate higher throughput. One approach we have pursued is machine learning [100,109]. This approach extracts and classifies different types of larvae based on their similarities in VMR patterns. These similar larvae likely share similar genetic information, or have been exposed to similar experimental conditions. In theory, machine-learning approaches can analyze larvae individually, which may substantially reduce the required sample number in experiments. We started testing this idea by using different machine-learning approaches to classify WT and *pde6c* mutants [109]. We found that one approach, the support vector machine, provided classification accuracy as high as 95%. We expect that this approach may facilitate analysis of individual larva in the future.

Together, these considerations and developments strongly position VMR as a promising in vivo approach to screen drugs for RD in a high-throughput manner. To realize this goal, other logistical issues in such high-throughput screens should also be considered.

## 7. Logistical Considerations in Using Zebrafish for High-Throughput Drug Screening

In this section, we will first consider how high-throughput assays are traditionally designed and conducted. Then, we will discuss how such screens have been done in vivo using zebrafish behavior, and what we have learned from these studies that may facilitate using zebrafish visual behavior to screen RD drugs.

### 7.1. Traditional Methods for High-Throughput Drug Screening

There are a number of methods to facilitate designing a drug screen [138]. Traditionally, assays are developed around biochemical or cell-based approaches [139,140,141], and the readouts are generally colorimetry, fluorescence, or luminescence [142,143,144]. In a cell-based approach, the readout could be monitoring the morphology of the cell and subcellular organelles. These approaches focus on targeting a molecule, such as a protein, or nucleotides that will interact with the drug. By focusing on a simple assay and readout, these target-based approaches allow for high-throughput screens that can result in approximately 50-million data points, with each point being the result of a single drug tested at a single concentration [145]. This maximizes the chances of a lead hit by screening a large number of compounds. However, these target-based, high-throughput techniques have two major drawbacks. First, they can only be performed by those with sophisticated screening facilities, typically pharmaceutical companies. Second, they may not reveal whether function of the disease model will be restored by the positive compounds. For example, a screen may find successful leads to prevent cells from dying, but it cannot guarantee that the surviving cells would function properly. In fact, target-based approaches are associated with a decline of efficacy in finding new drugs [146,147]. Hence, scientists are keen to find additional efficient approaches.

An alternative drug-screening approach would be to find a lead that can restore function or alleviate disease phenotype in a whole animal. This in vivo, phenotype-based screening was the predominant screening approach before the advent of high-throughput techniques [148]. Using the previously described cell-death example, a lead hit from phenotype-based screening would keep the dying cell alive, while restoring functions that are altered by the cell death. One major advantage of this approach is that a screen can take place before the disease and drug mechanisms are elucidated [149]. For example, the anticonvulsant Levetiracetam was discovered in 1992 during a random chemical screen to prevent seizures in mice. Yet, to this day, the molecular mechanism of Levetiracetam remains unknown [150].

### 7.2. Drawbacks of Using Zebrafish Behavior for Drug Screening

The phenotype-based approach using zebrafish also has some drawbacks compared to the contemporary high-throughput-screening technologies. In general, a screen based on zebrafish behavior will take more time to complete than a traditional screen, as we need to control many factors that can influence the behavior of larval zebrafish. For example, they typically need to acclimate to a new environment, such as a 96-well plate, or a testing chamber. Without doing this, their behavior may vary, which would in turn affect the sensitivity of the assay. When we worked with RD zebrafish larvae, we needed to acclimatize them, taking several hours for us to finish the whole assay. However, slower throughput is relative; such screens may take even longer to perform if they are done with mice models. In addition, light perception of zebrafish is controlled by circadian rhythm [151]. Therefore, screening with zebrafish cannot generally be performed all hours of the day without sophisticated scheduling and groupings of larvae into photoperiods. There are also some variations in locomotor between wild-type (WT) strains and within clutches [101,111,113,114]. These variations are likely originated from the genetic differences between WT strains [152,153], and between individuals within a strain. Thus, it is advisable to use one particular WT strain for any study, a consideration that will hold true for any other vertebrates used in phenotype-based in vivo screens for eye drugs. With all of the advantages and disadvantages taken together, zebrafish visual-behavioral assays have significant potential to identify novel therapeutics that can functionally treat RD.

While zebrafish behavioral assays may identify functional drugs, they do not provide information on the effects occurring at the tissue level or below. Thus, many zebrafish drug-screening assays also utilize imaging techniques for primary data acquisition. For example, transgenic zebrafish expressing fluorescent proteins in the developing vasculature can be used for screening compounds that affect angiogenesis [154]. Another example is that researchers are applying Förster resonant energy transfer (FRET) techniques in zebrafish to gain insight into the biochemical aspects of disease [155,156]. Since a positive lead from cellular/biochemical data may or may not translate into effective systemic treatment, a lead from a behavioral screen will likely be more successful as the disease phenotype is remediated at the functional level. Therefore, a behavioral screen should be validated by cellular and biochemical imaging techniques to ensure robustness of the treatment, and may potentially provide insight into the molecular mechanism.

This behavioral-screen strategy may identify new drugs for RD, where underlying genetic basis and pathogenesis of many subtypes are still unclear. These conditions may not have definitive molecular targets for biochemical screens, making such an endeavor inefficient and risky. On the contrary, they can be benefited by behavioral phenotype-based screening, as this approach does not require a specific molecular target to begin with. Between 1999 and 2008, phenotype-based drug screening identified 37% of the first-in-class drugs [148] which have a novel mechanism of action for treating a disease that no other current drugs have targeted. We believe that new drugs can be found by behavioral screen through restoring visual function to RD zebrafish models.

### 7.3. Auxiliary Technologies to Facilitate High-Throughput Drug Screening in Zebrafish

During high-throughput screens in zebrafish, a few logistical issues may arise and must be addressed. One major hurdle is being able to routinely collect enough embryos for screening. This number can be several thousand on a daily basis. This is achievable in the fish model because each pair of sexually-mature adults can produce up to 200 eggs on a weekly basis. As these adults are small (~1–2 inches), many adults can be housed in a moderate size facility and provide the desired number of embryos. However, breeding these adults efficiently represents another hurdle. They are usually bred in small static tanks in pairs, or in small groups for collecting several hundred embryos. As a screen is scaled up, using many small breeding tanks becomes inconvenient and too labor-intensive. To address this hurdle, a single, large breeding vessel should be used for breeding many fish. This approach takes advantage of the group-breeding behavior of zebrafish and allows them to breed in their preferred way. Such large-scale breeding systems are available from several suppliers: Pentair Aquatic Eco-Systems [157], Techniplast [158], and Aquaneering Incorporated [159]. Their performance was evaluated by a study that used a similar home-made device that was able to generate over 8000 embryos in a breeding session [160]. This device was much more efficient than 40 smaller tanks for breeding the same number of fish. It not only significantly reduced the breeding time, but also produced twice as many embryos.

Another hurdle in efficient drug screening is the handling of large numbers of embryos. In many current screens, the embryos are manually transferred. In large-scale screens, they can be automatically sorted and dispensed. This can be achieved by microfluidics, an engineering approach that controls small volume of fluids. This microfluidics approach has been applied to high-throughput zebrafish research, with great success [161,162]. One of these systems has been developed by an applied and industry-commissioned research and development organization, CSEM [163]. This CellFactor system can sort and dispense zebrafish eggs 96-well plate in less than 7 min. Another system, vertebrate automated screening technology (VAST) [161], is commercialized by Union Biometrica [164] as the VAST BioImager^TM^. This platform can automatically take up, orient, and image individual zebrafish larvae on the attached microscope, and can dispense the larvae in a multi-well plate. By attaching to a fluorescent microscope, this system can inspect transgenic fish tagged with fluorescent proteins. This can greatly facilitate sorting and isolating of visual mutants that are tagged with specific reporters. For example, the Q344X transgenic mutant [59], discussed above, carries a GFP reporter that expresses in the olfactory bulbs. The reporter can be used to quickly isolate and verify all transgenic mutants in the automated-sorting system for drug screening. In the original study of VAST system, the authors screened for an *astray/robo2* mutant with defects in retinal axon guidance. They could distinguish WT from *astra/robo2* mutants, with a sensitivity of 100% and specificity of 98.8% for a 96-well plate with 83 randomly seeded mutants. They estimated that a complete cycle of loading, positioning, cellular-resolution imaging and dispensing a larva would take less than 16 s.

## 8. Conclusions

In this review, we have evaluated how to use zebrafish for in vivo screening of RD drugs. The zebrafish is an excellent vertebrate model for eye-disease research and high-throughput studies. Its visual behavior VMR can be compatible with high-throughput drug screening. We believe that future developments and optimizations of the VMR assay could expedite discovery of new RD drugs. These drugs may treat the incurable RD, prolong the vision of visually-impaired patients, and give them hope for the future.

## Figures and Tables

**Table 1:** Recent in vivo zebrafish screens.

Zebrafish Drug Screening Study	Number of Compounds Screened	Number of Reported Hits	Reference
Bruni et al. (2016)	24,760	Top 100	[69]
Dinday et al. (2015)	1000	4	[70]
Gallardo et al. (2015)	2960	165	[71]
Li et al. (2015)	3120	4	[72]
Nath et al. (2015)	13,120	1	[73]
Liu et al. (2014)	3000	8	[74]
Jin et al. (2013)	1200	6	[75]
Kokel et al. (2013)	10,000	1 Pursued	[76]
Nath et al. (2013)	3120	4	[77]
Baxendale et al. (2012)	2000	46	[78]
Kokel et al. (2010)	14,000	1627	[68]
Rihel et al. (2010)	5648	547	[67]

Zebrafish are being utilized to screen drugs for a variety of topics. This table shows example screens that have recently been completed, the number of drugs that were screened, and the number of reported hits according to the criteria defined by the investigators of the study.
